# Effects of simulated winter short photoperiods on the microbiome and intestinal metabolism in Huanghe carp (*Cyprinus carpio haematopterus*)

**DOI:** 10.3389/fendo.2023.1293749

**Published:** 2024-01-04

**Authors:** Wenqian Wang, Shengyan Su, Ping Dong, Wenrong Feng, Jianlin Li, Chengfeng Zhang, Yongkai Tang

**Affiliations:** ^1^ Wuxi Fisheries College, Nanjing Agricultural University, Wuxi, China; ^2^ Key Laboratory of Freshwater Fisheries and Germplasm Resources Utilization, Ministry of Agriculture and Rural Affairs, Freshwater Fisheries Research Center, Chinese Academy of Fishery Sciences, Wuxi, China

**Keywords:** *Cyprinus carpio*, seasonal photoperiod, microbiota, metabolism, aquaculture

## Abstract

**Objective:**

As one of the most important environmental signals, photoperiod plays a crucial role in regulating the growth, metabolism, and survival of organisms. The photoperiod shifts with the transition of the seasons. The difference in photoperiod between summer and winter is the greatest under natural conditions. However, the effect of photoperiod on Huanghe carp (*Cyprinus carpio haematopterus*) was paid little attention. We investigated the impact of artificial manipulation of seasonal photoperiod on Huanghe carp by integrating growth performance, intestinal flora, and intestinal metabolome.

**Method:**

We conducted an 8-week culture experiment with summer photoperiod (14 h light:10 h dark, n = 60) as the control group and winter photoperiod (10 h light:14 h dark, n = 60) based on the natural laws.

**Results:**

Winter photoperiod provokes significant weight increases in Huanghe carp. The altered photoperiod contributed to a significant increase in triglyceride and low-density lipoprotein cholesterol levels and the gene expressions of lipid metabolism in the intestine of Huanghe carp. 16s rDNA sequencing revealed that winter photoperiod diminished intestinal flora diversity and altered the abundance. Specifically, the relative abundances of Fusobacteria and Acidobacteriota phyla were higher but Proteobacteria, Firmicutes, and Bacteroidetes phyla were reduced. Analogously, photoperiodic changes induced a significant reduction in the *Pseudomonas*, *Vibrio*, *Ralstonia*, *Acinetobacter*, and *Pseudoalteromonas* at the genus level. Additionally, metabolomics analysis showed more than 50% of differential metabolites were associated with phospholipids and inflammation. Microbiome and metabolome correlation analyses revealed that intestinal microbe mediated lipid metabolism alteration.

**Conclusion:**

The winter photoperiod induced intestinal flora imbalance and lipid metabolism modification, ultimately affecting the growth of Huanghe carp. This study provides new insights into the effects of seasonal photoperiodic alteration on the well-being of fish.

## 1 Introduction

The rotational and translational movement of the Earth triggers variations in luminance that fluctuate over a 24-hour or seasons of the year period (1). Most organisms exhibit rhythms in their physiology and behavior. Since identical fish can exhibit circadian behaviors, the circadian rhythm system in fish exhibits profound flexibility (2). Evidence suggested that fish circadian mechanisms include photosensitive central nervous system-related clock organs, peripheral photosensitive tissues with autonomic circadian clocks, and entrainable circadian oscillators. The structurally and functionally complex fish circadian system maintains coordination in order to ensure physiological adaptation to a periodically changing environment (3). Photoperiod is an important factor for fish, involving numerous biological processes such as growth performance, foraging, and survival. Hence, photoperiod is also significant in the long-term cultivation of artificial aquaculture systems.

The gut microbiota has been identified as an environmental factor that significantly impacts digestion absorption and physiological metabolism in animals (4, 5). Its composition and proportions are dynamic, depending on both internal and external factors of the host (6). Different photoperiod regimes have the capacity to shape the microbiota (7–9). Bailey et al. demonstrated that prolonged daylight significantly enhanced the relative abundance of bacteria in the phylum Proteobacteria (10). Reproductive hormone and gene expression levels as well as the gut microbiota of voles (*Lasiopodomys brandtii*) were also impacted by long-day and short-day photoperiod (11). In a study simulating seasonal changes in light and dark cycles, the ratio of Firmicutes to Bacteroidetes in the cecum and large intestine was significantly higher in winter-simulated conditions than in summer-simulated conditions (9). Wu et al. found that continuous darkness suppressed gut microbial rhythmic oscillations and induced a concomitant increase in the abundance of Clostridia, suggesting that light is a necessary factor for the normal rhythm of intestinal flora (12). Photoperiod manipulation not only affects mammalian gut flora, but also strongly shapes the microbial community in fish. Under extended darkness, gut microbial changes induced impair lipid metabolism in the gut of zebrafish (*Danio rerio*) and provoke apoptosis, compromising fish health (8). Given this, we speculate that photoperiod may influence carp physiological health by adjusting the composition of the gut microbiota, but such studies are still lacking.

The common carp (*Cyprinus carpio*) is one of the most widely distributed freshwater fish species with high species diversity. Total production of carp aquaculture reached 4.2 million tons (Mt) in 2020, accounting for 8.6% of the world’s total aquaculture production (13). Huanghe carp (*Cyprinus carpio haematopterus*) is preferred by consumers because of its profound historical and cultural heritage (14). Photoperiod, as one of the most important environmental signals, impacts organism growth, metabolism, and survival (15, 16). However, the effect of photoperiodic changes was easily ignored on carps in experimental studies and culture. The difference in photoperiod between summer and winter is the greatest under natural conditions, and we were intrigued by the impact of photoperiod on carp in addition to temperature. Therefore, we selected experimental conditions with current seasonal [summer; 14 h light (L):10 h dark (D)] and winter (10L:14D) photoperiods to investigate the effect of seasonal photoperiod changes on the intestinal metabolism of Huanghe carp using gene variations, 16s rDNA sequencing, and metabolomic analyses.

## 2 Materials and methods

### 2.1 Animals and experimental design

A total of 120 juvenile Huanghe carp (6.5 ± 1.5 g) were selected from the Freshwater Fisheries Research Center, Chinese Academy of Fishery Sciences, and cultured in laboratory tanks for three weeks, after which they were transferred to tanks for acclimatization in the formal experiment for one week (14.5 ± 3 g). The entire experiment was conducted during the summer months. In order to conduct the protocols, the fish were kept in indoor 300-L tanks (20 fish per tank) with constantly aerated water in a recirculating system (temperature 24–28°C, pH 7.2–7.8, dissolved oxygen > 6 mg L^−1^). Fish were daily fed three times with the refined freshwater fish No. 1 compound feed (crude protein level 35%, crude fat level 3%) from Ningbo Tech-Bank Co., Ltd., slowly until there were no more fish feeding, and the residual bait and feces were removed.

Fish were divided into two groups: the control group (Con, n = 60), which was exposed to a summer lighting cycle (14L:10D); winter (short-period) photoperiod (SP, n = 60), which simulated the sunlight time rhythm of winter pattern in China (10L:14D). Each group had 3 replicates of 20 fish each. Fish were randomly allocated into the experimental tanks. A white light-emitting diode light (main peak 460 nm), simulating the natural light, was hung above the tank. The light intensity was 800 ± 20 luxes in all treatments during the whole experiment. To avoid interference, each tank was covered by light proof black material. In the eighth week of the trial, 10 fish per culture barrel were randomly selected (n = 30/group), euthanized with MS-222 (100 mg L^−1^), and weighed. Subsequently, these fish were dissected on a sterile bench to collect intestine samples for intestinal microbiota determination and metabolome analysis. All samples were immersed in liquid nitrogen and stored in an ultrafreezer (−80°C) for subsequent examination.

### 2.2 Biochemical parameters of intestinal tissues

We collected intestinal samples of Huanghe carp for measuring parameters (n = 9/group), including triacylglycerol (TG), total cholesterol (CHOL), and low-density lipoprotein cholesterol (LDL-C) according to the manufacturer’s instructions. All the assay kits were purchased from Nanjing Jiancheng Bioengineering Institute, China. In detail, triacylglycerol and total cholesterol were determined by the GPO-PAP enzyme method, low-density lipoprotein cholesterol was determined by the microplate method.

### 2.3 16S rDNA amplicon sequencing analysis

Gut microbial analysis was performed using 16S rDNA amplicon sequencing to amplify the V4 variable regions of the 16S rRNA gene. Genomic DNA was first extracted from 12 intestinal samples using the CTAB/SDS method (n = 5/group). After detection, PCR amplification and product purification were performed, followed by library construction. Library quantification was followed by up-sequencing using Illumina NovaSeq 6000. The final Amplicon Sequence Variants (ASVs) were obtained by splicing, filtering, and noise reduction for the double-ended reads obtained from sequencing. Species annotation was performed using the classify-sklearn algorithm for each ASV using the naive bayes classifier to obtain the corresponding species information and species-based abundance distribution. The number of sequences of each sample at each taxonomic level (kingdom, phylum, order, family, genus, species) was counted based on the annotation results of ASVs and the characteristic table of each sample. Based on the species annotation results, the top ten species with maximum relative abundance at the taxonomic level of phylum and genus were selected for analysis for each sample. Alpha diversity was calculated using QIIME2 software (v2021.2), and beta diversity was assessed by principal coordinate analysis (PCoA) based on the calculation of Unifrac distance. *P* < 0.05 was considered a statistically significant difference. The original data analysis was completed by Novogene Co., Ltd. (Beijing, China).

### 2.4 LC/MS untargeted metabolomic analysis

Non-targeted metabolomic analysis using 12 intestinal samples was done on a liquid chromatography-mass spectrometry (LC-MS) (n = 6/group). Metabolites in intestinal tissue were extracted with 1000 μL of a pre-cooled mixture of methanol, acetonitrile, and water (v/v/v, 2/2/1), and then detected by LC-MS/MS. Detect as many molecular characteristic peaks in the sample as possible based on high-resolution mass spectrometry (HRMS) detection technology. Combined with the high-quality mzCloud database constructed from standard materials, mzVault and MassList databases, the molecular characteristic peaks were matched and identified. Spectral processing and database search were performed using Compound Discoverer software (3.1) to obtain qualitative and quantitative results of the metabolites, and then quality control was performed to ensure the accuracy and reliability of the data results. The data were transformed and subjected to partial least squares discriminant analysis (PLS-DA). Hierarchical cluster analysis (HCA) and metabolite correlation analysis were performed on all the differential metabolites obtained. The identified metabolites were annotated using the KEGG database. The screening criteria for differential metabolites were VIP > 1, *P* < 0.05. The original data analysis of omics was completed by Novogene Co., Ltd. (Beijing, China).

### 2.5 RNA extraction and quantitative real-time PCR

Total RNA from the intestine was extracted with TRIzol reagent (Vazyme Biotechnology Co., LTD., Nanjing, China) (n = 12/group). The quality and quantity of the extracted RNA were evaluated by the OD values and their ratios at 260 and 280 nm (1.8-2.1 for A260/A280). cDNA was synthesized using 1 μg of extracted RNA through a 20 μl reverse transcription reaction system using the HiScript III RT SuperMix for RT-qPCR (+gDNA wiper) kit (Vazyme). RT-qPCR was performed in a 20 μL reaction system containing specific primers and SYBR premixes (Vazyme), and cycling conditions as follows: 95°C pre-denaturation was performed for 30 s, followed by 40 cycles of 95°C for 5 s and 59–62°C for 1 min. Amplification was performed in a Thermal Cycler Dice Real Time System TP800 system. The final concentration of primers used for RT-qPCR was 0.4 μM. We used melting curves to quantify and assess the specificity of their RT-qPCR, and single peaks appeared, which indicated amplicons were specific for the genes. We used No Template Control and Endogenous Control as negative and positive controls to ensure that there was no contamination. Using *gapdh* as an internal reference (17), the expression levels of target genes were normalized. The expression of relevant RNA was analyzed by 2^-ΔΔCT^. The primer information of the selected genes is shown in **Table 1**. Primers for RT-qPCR were designed using primer premier 5.0.

**Table 1:** Primers used for real-time quantitative PCR (RT-qPCR) analysis of gene expression.

Gene	Primer sequence (5’ – 3’)	Amplification size (bp)
*rev-erbα*	F: CCCCTGCTCTTCCACTTCA	219
	R: GCGGCGATGGTTTTGTCT	
*nfil3*	F: TCGGTCATCAAGCACTCA	201
	R: CCCAGGAAAAGGACTACTAAGG	
*Acsl1*	F: CATCTTGACTATGGCAGCG	166
	R: GGCATAAGAGGGGAGGTAA	
*scd*	F: CTTCCTGTTTCCATCCGC	223
	R: CGAGTATTTGTGGTGAACGC	
*elovl1*	F: TACACCTGGAGATGTGACCT	220
	R: CCGCAGGAGTAAGAGTGA	
*c/ebpγ*	F: AGCCGTAAAGAAGAGCCG	173
	R: TGTCTGCGAGGTTGTGAGC	
*gapdh*	F: CCGTTCATGCTATCACAGCTACACA	310
	R: GTGGATACCACCTGGTCCTCTG	

Details of primer sequence and amplification size are included.

### 2.6 Statistical analysis

Based on the Spearman correlation coefficient, correlation analysis of the significantly different metabolites was performed based on the LC/MS non-targeted metabolomic analysis. Integrated analysis between differential metabolites and different bacteria was conducted to measure the correlation between species diversity and metabolites in intestinal samples. The range of the correlation coefficient is (−1, +1).

All statistical analyses were performed using IBM SPSS Statistics for Windows, version 27.0 (IBM Corp., Armonk, N.Y., USA). Kolmogorov-Smirnov and Levene’s test were utilized to test the normality of distribution of the data and homogeneity of variances, respectively. Student’s *t*-test was used to test the significance between the Con and SP groups in cases where the data obeyed a normal distribution and exhibit homoscedasticity. Mann-Whitney U-test was used when the data did not conform to normal distribution or homoscedastic. *P* < 0.05 was considered statistically significant. Data were expressed as the mean ± standard error (SEM).

## 3 Results

### 3.1 Disturbances in intestinal lipid metabolism in Huanghe carp caused by seasonal photoperiod

**Figure 1:**
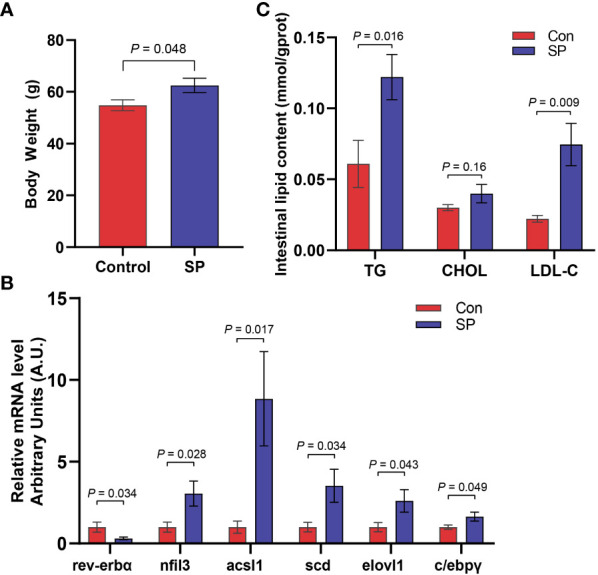
Seasonal light interferes with the circadian rhythm of Huanghe carp and causes intestinal lipid metabolism disorder. **(A)** Body weight (g) in Con and SP groups (n = 30/group). **(B)** Expression of genes associated with circadian rhythm and lipid metabolism in intestine (n = 12/group). **(C)** Levels of TG, CHOL, and LDL-C in the intestine of carp in Con and SP groups (n = 9/group). *rev-erbα*, nuclear receptor subfamily 1; *nfil3*, nuclear factor interleukin-3-regulated protein; *acsl1*, acyl-CoA synthetase long chain family member 1; *scd*, stearoyl-CoA desaturase; *elovl1*, elongation of very long chain fatty acids protein 1; *c/ebpγ*, CCAAT/enhancer-binding protein gamma; TG, triglyceride; CHOL, cholesterol; LDL-C, low-density lipoprotein cholesterol. The values are mean ± SEM.

Compared with the Con, the carp in SP group had a significant increase in body weight (*P* = 0.048) (**Figure 1A**). Seasonal photoperiodic exposure altered the expression of genes related to intestinal rhythm and lipid metabolism in carp, such as a significant up-regulation of *nfil3* (nuclear factor interleukin-3-regulated protein) (*P* = 0.028) and *rev-erbα* (nuclear receptor subfamily 1) (*P* = 0.034) was significantly decreased in SP (**Figure 1B**). It has been shown that *nfil3* is negatively regulated by upstream biological clock signaling (*rev-erbα*) and further affects intestinal lipid metabolism (18), so we examined the lipid levels in the intestine. The SP group showed significantly higher expression of intestinal adipogenesis-related genes including *acsl1* (acyl-CoA synthetase long chain family member 1) (*P* = 0.017), *scd* (stearoyl-CoA desaturase) (*P* = 0.034), *elovl1* (elongation of very long chain fatty acids protein 1) (*P* = 0.043), and *c/ebpγ* (CCAAT/enhancer-binding protein gamma) (*P* = 0.049) (**Figure 1B**). Similarly, TG (*P* = 0.016) and LDL-C (*P* = 0.009) levels were significantly higher in the intestine of Huanghe carp under winter photoperiod, but there was no significant difference in CHOL (**Figure 1C**).

### 3.2 Effect of seasonal photoperiod on the diversity of gut flora of Huanghe carp

**Figure 2:**
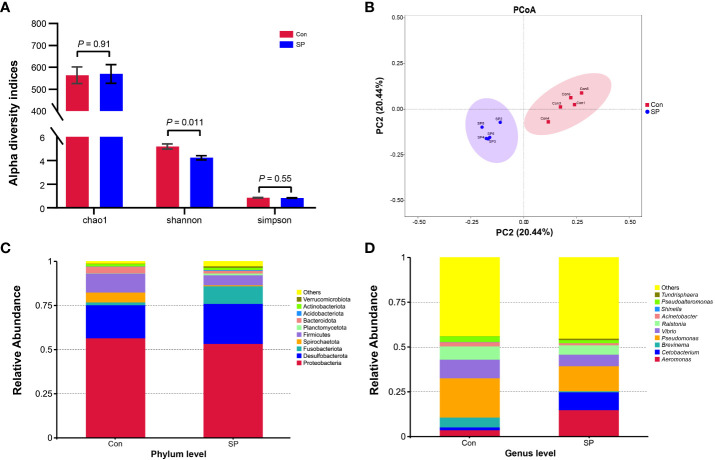
Effects of seasonal photoperiod on the gut microbiota diversity of Huanghe carp. **(A)** Alpha diversity indexes results. **(B)** PCoA results. **(C)** Relative abundance of species at the phylum level. **(D)** Relative abundance of species at the genus level. The values are mean ± SEM, n = 5/group.

Experiments were conducted to assess the composition and diversity of the gut microbial community of Huanghe carp under seasonal photoperiod using high-throughput sequencing technology. To evaluate the differences in alpha diversity among the groups, we analyzed chao1, shannon, and simpson indicators (**Figure 2A**). Changes in photoperiod significantly altered the shannon index compared to Con (*P* = 0.011). Principal coordinate analysis (PCoA) was further performed to assess the degree of similarity of microbial communities among the groups (**Figure 2B**). The results showed that the microbial community composition of SP was significantly different from that of the Con. As shown in **Figures 2C, D**, the microbial abundance differed between the Con and SP groups at the phylum and genus levels, respectively. And both groups have similar dominant flora. At the phylum level, Proteobacteria, Fusobacteria, and Firmicutes were detected as the dominant phyla in carp intestine (**Figure 2C**). At the genus level, *Aeromonas*, *Cetobacterium*, *Brevinema*, *Pseudomonas*, *Vibrio*, and *Ralstonia* were the dominant genera in the intestine of Huanghe carp (**Figure 2D**).

**Figure 3:**
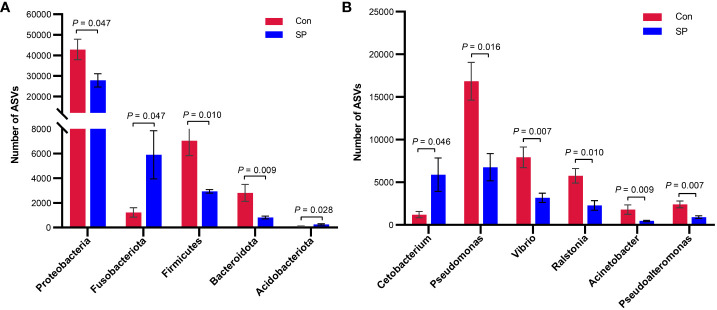
Phylum and genus level differential flora due to seasonal photoperiod. **(A)** Screened bacteria with significant differences by phylum level. **(B)** Screened bacteria with significant differences by genus level. ASVs, Amplicon Sequence Variants. The values are mean ± SEM, n = 5/group.

More specifically, we compared the abundance of different bacteria in the SP group with that of Con in **Figure 3**. At the phylum level (**Figure 3A**), photoperiod alteration significantly increased the abundance of Fusobacteria and Acidobacteriota phyla and decreased the abundance of Proteobacteria (*P* = 0.047), Firmicutes (*P* = 0.01), and Bacteroidetes phyla (*P* = 0.009). At the genus level (**Figure 3B**), the abundance of *Pseudomonas* (*P* = 0.016), *Vibrio* (*P* = 0.007), *Ralstonia* (*P* = 0.01), *Acinetobacter* (*P* = 0.009), and *Pseudoalteromonas* (*P* = 0.007) was significantly reduced, while at the same time the number of *Cetobacterium* (*P* = 0.046) was significantly increased.

### 3.3 Seasonal photoperiod triggers metabolite changes in the intestine of Huanghe carp

**Figure 4:**
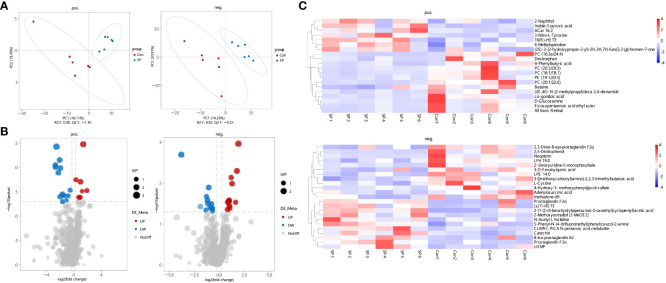
Characteristics of gut metabolites between the Con and SP groups (n = 6/group). **(A)** PLS-DA scores plots. **(B)** Volcanic map of differential metabolites. Red represents the up-regulation; blue represents the down-regulation; gray represents no significant change. **(C)** Hierarchical cluster analysis of differential metabolites. pos. and neg. stand for positive and negative ion modes applied to detection analysis.

In conjunction with the previous findings, we performed non-targeted metabolomic analyses of carp intestinal samples exposed to seasonal photoperiod to further explore the effects of seasonal photoperiodic changes on the gut. To maximize the metabolite coverage, both positive ion (pos) and negative ion (neg) modes were used in the assay analysis. According to the PLS-DA plot, it can be shown that metabolites are largely separated between SP and Con (**Figure 4A**). Under simulated winter light duration conditions, 43 differential metabolites (DMs) were screened of which 18 were up-regulated and 25 were down-regulated (**Figure 4B**). Additionally, these 43 DMs separated positive and negative metabolites were clustered into different subclusters according to their relative content in the heatmap (**Figure 4C**).

**Figure 5:**
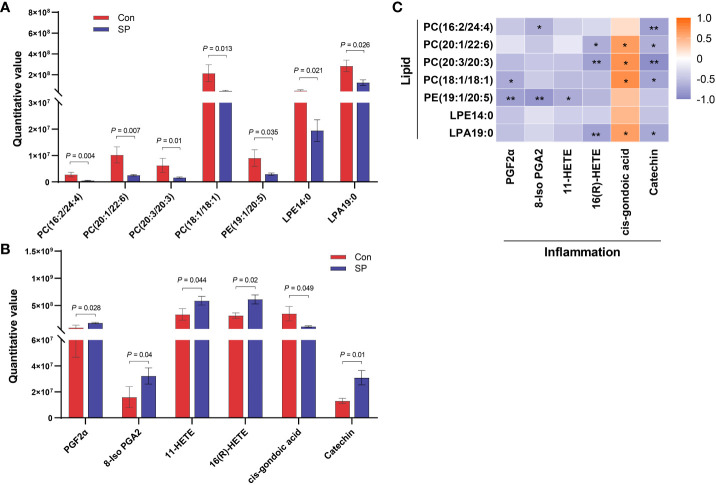
Correlation analysis of intestinal differential metabolites in Huanghe carp between Con and SP groups. **(A)** Phospholipid-related differential metabolites. **(B)** Inflammatory-related differential metabolites. **(C)** Spearman correlation between phospholipid related metabolites and inflammation related metabolites under winter (short-period) photoperiod. PC, phosphatidylcholine; PE, phosphatidylethanolamine; LPA, lysophosphatidic acids; LPE, lysophosphatidylethanolamine; PGF2α, Prostaglandin F2α; 8-Iso PGA2, 8-Iso prostaglandin A2. The values are mean ± SEM, n = 6/group. *P < 0.05, **P < 0.01.

We observed DMs induced by winter light mainly associated with phospholipids and inflammation (**Figure 5**). We found significant reductions in phospholipid-related metabolites, including phosphatidylcholine (PC), phosphatidylethanolamine (PE), lysophosphatidic acids (LPA), and lysophosphatidylethanolamine (LPE) (*P* < 0.05) (**Figure 5A**). This suggests that the reduction in light duration altered phospholipid levels and triggered abnormal lipid metabolism in intestine. Notably, arachidonic acid metabolism intermediates such as Prostaglandin F2α (PGF2α) (*P* = 0.028), 8-Iso prostaglandin A2 (8-Iso PGA2) (*P* = 0.04), 11-HETE (*P* = 0.044), and 16(R)-HETE (*P* = 0.02) were upregulated (**Figure 5B**). Subsequently, we assessed whether photoperiodically altered carp intestinal lipid-related metabolites were associated with inflammation-related metabolites. Spearman correlation analysis revealed that PCs, PE, and LPA were negatively correlated with most of the inflammation-related metabolites such as PGF2α, 8-Iso PGA2, 11-HETE, and 16(R)-HETE. The opposite was cis-gondoic acid, which revealed a positive significant correlation with the lipid-related metabolites, totally opposite to the rest of inflammation-related metabolites (**Figure 5C**).

### 3.4 Correlations between intestinal microbiota and metabolome

**Figure 6:**
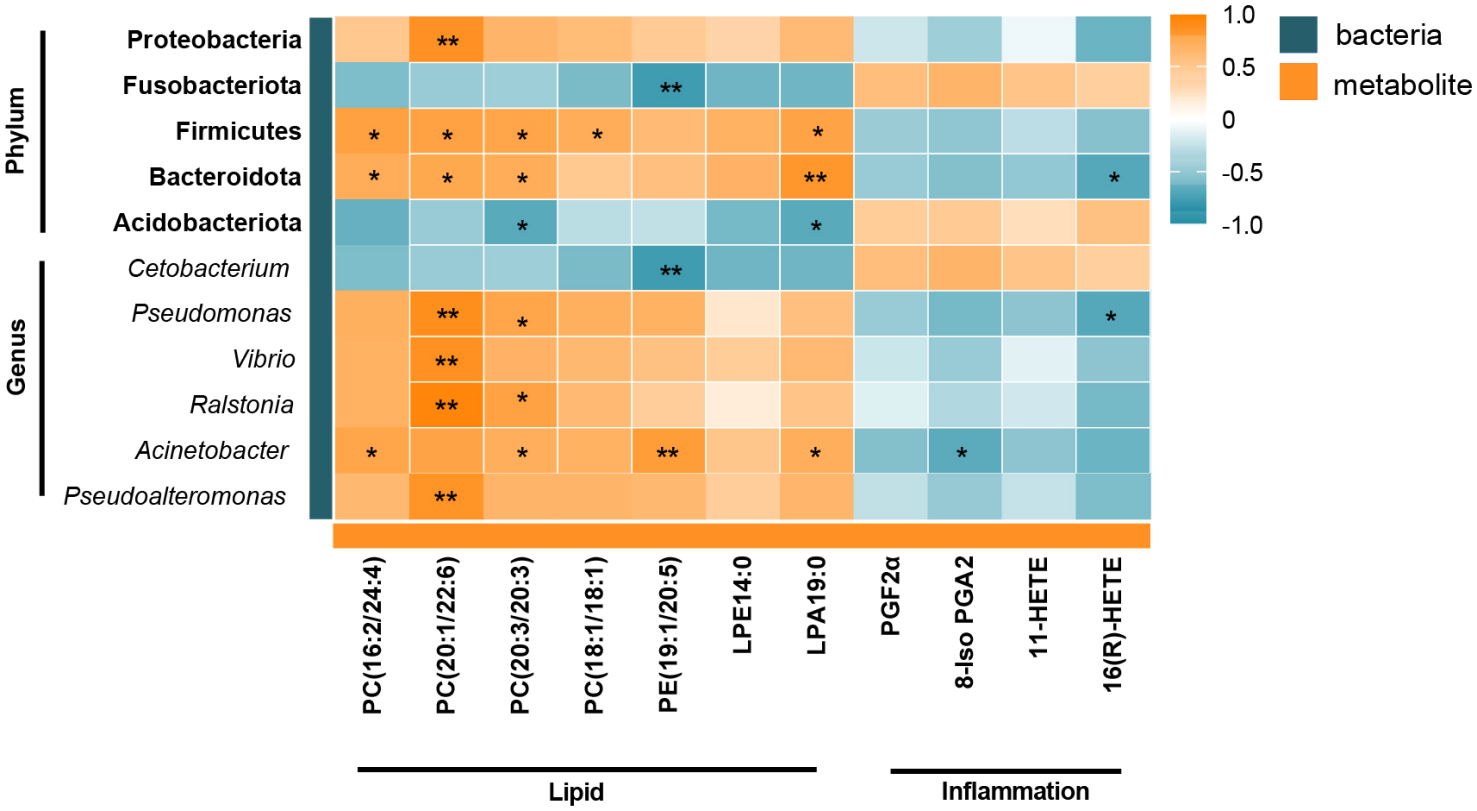
Heatmap of correlation between the gut differential metabolites and differential microbiota under the influence of winter (short-period) photoperiod. Orange indicates positive correlations and green indicates negative correlations. *P < 0.05, **P < 0.01.

Correlation analysis was used to analyze the correlation between differential flora and lipid- and inflammation-related DMs to determine the relationship between intestinal metabolites and microorganisms. As shown in **Figure 6**, Firmicutes and Bacteroidetes were positively correlated with PC(16:2/24:4), PC(20:1/22:6), PC(20:3/20:3), and LPA19:0 at phylum level. Whereas PC(18:1/18:1) was only positively correlated with Firmicutes, but not with Bacteroidetes. Additionally, there was a highly positive correlation between Proteobacteria and PC(20:1/22:6), Fusobacteriota was significantly negatively correlated with PE(19:1/20:5), and Acidobacteriota was negatively correlated with PC(20:3/20:3) and LPA19:0. At the genus level, PC(20:1/22:6) was significantly positively correlated with *Pseudomonas*, *Vibrio*, *Ralstonia*, and *Pseudoalteromonas*, and PC(20:3/20:3) was positively correlated with *Pseudomonas*, *Ralstonia*, and *Acinetobacter*. PE(19:1/20:5) was positively correlated with *Acinetobacter* and significantly negatively correlated with *Cetobacterium*. Moreover, arachidonic acid metabolism intermediate 16(R)-HETE had a markedly negative correlation with Bacteroidetes and Pseudomonas. Overall, Firmicutes, Bacteroidetes, and *Acinetobacter* were the shared flora for metabolites PC and LPA. Most of the intestinal flora and inflammatory metabolites had no obvious direct correlation, but we found an association through the correlation of lipid-related metabolites and inflammation-related metabolites (**Figure 5C**).

## 4 Discussion

Annual variation of photoperiod is the temporal organization of seasonal activities in the biological community, which plays an important role in regulating physiological processes such as growth, reproduction, and sex determination in fish (19, 20). Periodic environmental changes coordinate biological functions through the complex circadian system of fish (3). The variation in *nfil3* and *rev-erbα* in the present study also indicated that photoperiodic changes contribute to the regulation of clock genes, which further affects lipid metabolism (18). Various studies have demonstrated that circadian rhythm disruption broadly affects host lipid metabolism (21–24). It is well documented that seasonal variation such as photoperiod affect lipid deposition in farmed fish as well as the metabolic status of fish (25). Similarly, we observed increased body weight and intestinal TG and LDL-C in the SP group, which is consistent with the study by Xie et al. (26). Also, the expression of intestinal lipid synthesis-related genes *acsl1*, *scd*, *elovl1*, and *c/ebpγ* were higher under intestinal winter photoperiod in comparison to the control group. Together, winter photoperiod enhanced the expression of lipid synthesis-related genes, and thus lipid accumulation may be the crucial reason for weight gain under winter photoperiod.

Dysbiosis of the intestinal flora interferes with fish growth and development, nutrient metabolism, and immune regulation, ultimately affecting fish health (27). Moreover, intestinal flora modulates intestinal epithelial development and stimulates the immune system to regulate fish physiological functions (27, 28). Gut microbes are highly susceptible to changes in the external environment. The external environment transforms the composition and diversity of fish intestinal flora, resulting in modifications of metabolic functions and thus affecting the growth of fish (29). To our knowledge, there are few reports on the effects of photoperiod on the gut microbes of aquatic animals. We observed a decrease in microbial diversity in the SP group. It was shown that the alpha diversity of fecal microbiota was significantly lower under a 6L:18D photoperiod compared to the normal 12L:12D cycle, suggesting that photoperiod helps to maintain a higher species of gut microbiota (30). However, short photoperiod exposure reduces the number of bacterial species and remodels the distribution of intestinal flora (31). Thus, it’s difficult to maintain higher number of gut bacteria species throughout the winter photoperiod, which mean that the intestinal flora homeostasis was disrupted, possibly mediating metabolic shifts in Huanghe carp.

In addition to diversity, the relative abundance of intestinal flora directly reveals the function of specific gut microbes. In general, under normal conditions, the composition of the intestinal flora tends to be dynamically balanced and relatively stable (32, 33). However, the winter photoperiod showed significantly different gut microbial composition. Photoperiodic changes caused the differences in the abundance of intestinal flora (8). Arreaza-Gil et al. found that photoperiodic effects exhibited a trend toward decreased relative abundance of Firmicutes and increased Bacteroidetes level under 18L:6D (30). In the present experiment, the winter photoperiod showed lower abundance of Firmicutes and Bacteroidetes. In fact, members of Firmicutes such as *Lactobacillus* are involved in various carbon sources fermentation (34). Additionally, Bacteroidetes contains some bacteria with fermentative function on carp intestinal food (35), acting mainly on the metabolism of polysaccharides and bile acids (36). Thus, the winter photoperiod affects the intestinal digestion of Huanghe carp. Moreover, our experiments revealed a significant increase in the abundance of Fusobacteria in SP. Elevated Fusobacteria produces lipopolysaccharides (LPS) and endotoxins, involving inflammatory responses in the intestine (37). At the genus level, most of the bacterial genera altered by photoperiod belong to Proteobacteria phyla. For example, the relative abundance of *Pseudomonas*, *Vibrio*, *Ralstonia*, *Acinetobacter*, and *Pseudoalteromonas* was significantly reduced. It has been shown that *Vibrio*, *Pseudomonas*, and *Acinetobacter* contribute to some extent to intestinal digestion in fish (35, 38). *Pseudomonas* as a potential probiotic has important functions on intestine health, and *Vibrio* is one of the cellulose degrading microorganisms from the intestine of grass carp (39). In a study on sea bass (*Dicentrarchus labrax*), *Acinetobacter*, *Pseudomonas*, and *Vibrio* genera in the gut produced lipases and proteases (40). MacDonald et al. reported that strains belonging to *Acinetobacter* and *Vibrio* in the gut microbiota of Dover sole (*Solea solea* L.) were able to degrade chitin (41). Thus, it is evident that the winter photoperiod adversely affects the intestinal flora by modifying digestion and metabolism of nutrients. These changes in flora structure may also be a trigger of the body weight gain under winter photoperiod.

Crosstalk between the gut microbiota and its host depends in part on the production of metabolites, which have a significant impact on host physiology (42). Arachidonic acid related metabolites and phospholipids such as PE, PCs, and LPA exhibited obvious variations in Huanghe carp under the winter photoperiod. Winter photoperiod decreased almost all of the phospholipid levels, indicating that phospholipid metabolism in the intestine of Huanghe carp is extremely sensitive to seasonal photoperiod. In the present study, bacteria such as Firmicutes, Bacteroidetes, and Fusobacteria phyla showed a significant correlation with PC and PE. PC is the main bioactive phospholipid in the intestine (43). Alterations in microbial communities may play an important role in PC utilization (44). However, PE is involved in mitochondrial respiration and autophagy. In addition to its role in membrane structure formation, PE as a substrate is implicated in specific biosynthesis such as PC (44, 45). PE is a major precursor of endogenous cannabinoids with regulatory intestinal barrier function and anti-inflammatory effects, and its reduced levels can have a negative impact on mitochondrial energy metabolism and impair cell survival and growth (46–48). Down-regulated Proteobacteria, Firmicutes and Bacteroidetes were positively correlated with many PCs. However, up-regulated Fusobacteria was negatively correlated with PE. The results demonstrate that the changes in phospholipid metabolism under winter photoperiod are inextricably linked to gut microbe alterations in Huanghe carp. Additionally, PC and PE were mostly negatively correlated with arachidonic acid metabolism intermediates such as PGF2α, 8-Iso PGA2, 11-HETE, 16(R)-HETE. PC hydrolysis leads to the formation of fatty acids such as arachidonic acid, which activates inflammatory pathways. Arachidonic acid produces prostaglandins, thromboxaneA2, lipoxygenases, leukotrienes, and other metabolic products through three metabolic pathways regulated by cyclooxygenases (COX), lipoxygenases (LOX) and cytochrome P450 (CYP450) (49). These products are widely involved in organ function maintenance, inflammatory response and other physiological processes (50). Therefore, it is hypothesized that winter photoperiod affects lipid metabolism to trigger the production of inflammatory mediators.

## 5 Conclusion

Our study has shown that artificially imposed winter photoperiod can significantly increase body weight and induce lipid metabolism disorder in Huanghe carp. The changes in intestinal lipid metabolism in carp may be closely related to intestinal flora and various metabolites. Winter photoperiod reduced the relative abundance of some bacteria on nutrient digestion and metabolism in fish, such as the Firmicutes and Bacteroidetes phyla, and *Pseudomonas*, *Vibrio*, and *Acinetobacter* genera. The decreased phospholipids such as intestinal PC and PE were associated with an increase in inflammatory mediators such as prostaglandins and intermediates of arachidonic acid metabolism.

## Data availability statement

The data presented in the study are deposited in the National Center for Biotechnology Information (NCBI) repository, accession number PRJNA982570.

## Ethics statement

The animal study was approved by Nanjing Agricultural University. The study was conducted in accordance with the local legislation and institutional requirements.

## Author contributions

WW: Conceptualization, Data curation, Formal Analysis, Methodology, Software, Validation, Visualization, Writing – original draft. SS: Conceptualization, Methodology, Software, Visualization, Writing – review & editing. PD: Investigation, Software, Validation, Writing – review & editing. WF: Formal Analysis, Project administration, Software, Writing – review & editing. JL: Data curation, Formal Analysis, Investigation, Software, Writing – review & editing. CZ: Formal Analysis, Software, Writing – review & editing. YT: Conceptualization, Funding acquisition, Methodology, Project administration, Resources, Supervision, Writing – review & editing.
